# Carotid Stenosis and Cognitive Function: An Update on Therapeutic Interventions

**DOI:** 10.7759/cureus.81908

**Published:** 2025-04-08

**Authors:** Jesús Endara-Mina, Cristopher-Josue Escudero, Kerly Carreño, Cesar Intriago, Rafael López-Carrera

**Affiliations:** 1 Ecuavolcan Research Group, Pontificia Universidad Católica del Ecuador, Quito, ECU; 2 Research, Hospital de Especialidades Eugenio Espejo, Quito, ECU; 3 Research, Hospital General IESS (Instituto Ecuatoriano de Seguridad Social) Sur, Quito, ECU; 4 Research and Academic Affairs, Larkin Community Hospital, South Miami, USA; 5 Medical School, Universidad Central del Ecuador, Quito, ECU

**Keywords:** angioplasty, carotid artery disease, cognitive function, endarterectomy, revascularization

## Abstract

Carotid stenosis (CS) is closely associated with cognitive decline, primarily affecting memory, attention, and executive function. This relationship is explained by mechanisms such as chronic cerebral hypoperfusion and asymptomatic microembolism. Interventions like carotid endarterectomy (CEA) and carotid artery stenting (CAS) have demonstrated potential benefits in restoring cerebral perfusion; however, outcomes are variable, particularly in domains such as executive function. These differences may be attributed to patient characteristics, the degree of stenosis, and the technique employed. Revascularization is more commonly associated with the stabilization of cognitive decline rather than the active improvement of cognitive function. CEA has shown superiority over CAS in promoting recovery of cerebral connectivity and hemodynamic stability. Improvements have been documented using instruments such as the Montreal Cognitive Assessment (MoCA), especially in patients with baseline cognitive impairment. Complications such as postoperative cognitive dysfunction (POCD) and hyperperfusion syndrome underscore the importance of appropriate patient selection, taking into account factors such as advanced age, hypertension, and bilateral stenosis. Biomarkers such as the neutrophil-to-lymphocyte and platelet-to-lymphocyte ratios are associated with a higher risk of postoperative cognitive deterioration. Imaging modalities, including functional magnetic resonance imaging, support evidence of functional recovery following CEA. Questions remain regarding the long-term benefits, optimal selection criteria, and predictive value of biomarkers, all of which represent key areas for future research.

## Introduction and background

Carotid stenosis (CS) refers to the narrowing of the carotid arteries that are responsible for supplying blood to the brain. This condition is primarily caused by the accumulation of fatty plaques (atherosclerosis), leading to reduced cerebral blood flow and an increased risk of cerebrovascular events. CS is associated with cognitive decline, particularly affecting key functions such as attention, memory, and executive functioning [1,2].

Cognitive decline involves a reduction in mental abilities, including memory, thinking, judgment, or reasoning. In the context of CS, this decline may be pre-existing or progressive and is closely linked to diminished cerebral perfusion, which impairs neuronal function [3].

Individuals with CS have a 2.4- to 5-fold increased risk of developing dementia of the Alzheimer type [4]. This association is attributed to pathophysiological mechanisms such as asymptomatic microemboli and chronic cerebral hypoperfusion. These mechanisms are especially relevant in older patients with concomitant vascular diseases, including coronary artery disease or peripheral artery disease [1,2].

Carotid revascularization procedures, such as carotid endarterectomy (CEA) and carotid artery stenting (CAS), aim to restore adequate cerebral blood flow. These interventions may improve cognitive function in certain patients, particularly in the early stages of CS [3]. However, cognitive outcomes vary depending on the degree of stenosis, baseline cognitive status, and vascular comorbidities.

Several studies have demonstrated that even mild degrees of CS are associated with poorer performance on neuropsychological assessments compared to healthy individuals [2]. Unstable plaques, characterized by large lipid cores and inflammatory features, increase embolic risk and contribute to cognitive decline by causing cumulative microinfarcts [4]. In patients undergoing revascularization, plaque composition, such as the presence of a stable fibrous cap, may also influence cognitive outcomes [5].

The cognitive impact of revascularization is heterogeneous, suggesting that the effectiveness of these interventions depends on appropriate patient selection as well as individual factors such as age and vascular comorbidities. Declines in attention, memory, and executive function have been observed even in patients with moderate stenosis, underscoring the potential value of earlier interventions to preserve cognitive function [6].

The objective of this study is to evaluate the impact of carotid revascularization on cognitive function in patients with CS, with a specific focus on the short- and long-term cognitive effects of CEA and CAS.

## Review

Methods

**Figure 1:**
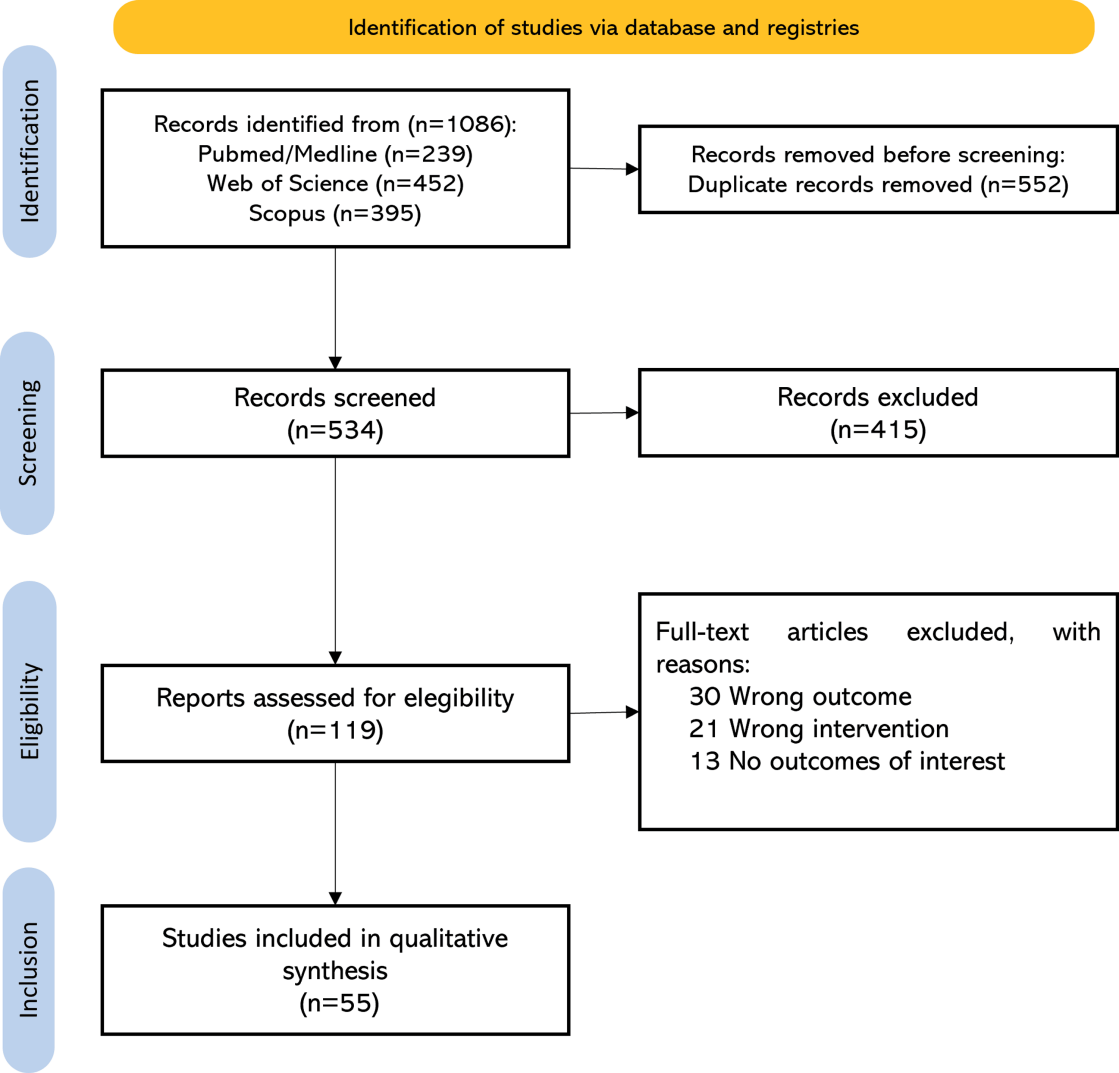
PRISMA flow diagram of study screening and selection PRISMA: Preferred Reporting Items for Systematic Reviews and Meta-Analyses

A narrative review was conducted to evaluate cognitive impairment in patients undergoing carotid revascularization. The search strategy followed the Preferred Reporting Items for Systematic Reviews and Meta-Analyses (PRISMA) guidelines [7], with details of the selection process presented in Figure 1. Literature searches were performed in Medline/PubMed, Scopus, and Web of Science. A combination of keywords related to carotid stenosis and neuropsychology was used to identify all relevant studies addressing cognitive assessment in the context of carotid revascularization. The search covered articles published between January 2018 and November 2024, focusing on this time frame due to recent advances in the field. Included materials comprised peer-reviewed original research articles, as well as narrative reviews, systematic reviews, and meta-analyses, with no language restrictions.

Keywords related to carotid revascularization and cognitive impairment included: “Cognitive changes,” “Cognitive impairment,” “Neurocognitive changes,” “Cognitive dysfunction,” “Carotid endarterectomy,” “Carotid artery surgery,” “Carotid artery stenting,” “Postoperative period,” “Postoperative,” and “Surgical outcomes.”

To address carotid stenosis within the search methodology, both general and specific terms were included, such as “Carotid stenosis,” “Carotid artery stenosis,” “Carotid artery narrowing,” and “Cerebrovascular stenosis.” More specific terms included “Internal carotid artery stenosis,” “Extracranial carotid artery stenosis,” “Bilateral carotid artery stenosis,” and “Unilateral carotid artery stenosis.” In addition, terms reflecting disease etiology were incorporated, including “Atherosclerotic carotid stenosis,” “Symptomatic carotid stenosis,” “Asymptomatic carotid artery stenosis,” “Severe carotid artery stenosis,” and “Moderate carotid artery stenosis.” Clinical context terms, such as “Carotid artery disease,” “Carotid plaque,” “Carotid artery occlusion,” and “Carotid atheroma,” were also considered.

Abstracts, conference posters, case reports, commentaries, and editorials were excluded from the analysis. The review process was conducted through a peer-review system, with an external reviewer involved in resolving any conflicts regarding article inclusion.

Physiopathology

**Figure 2:**
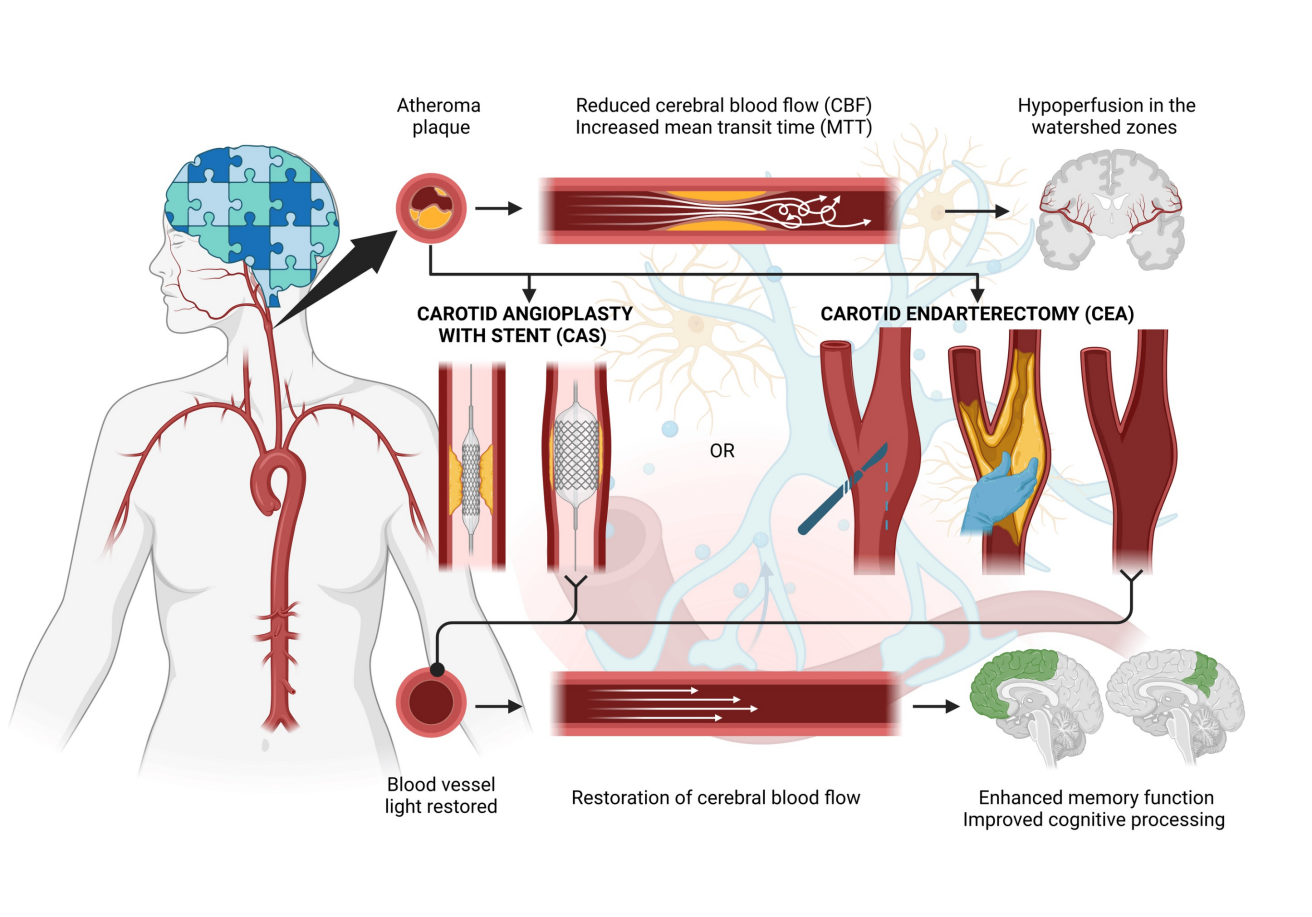
Pathophysiology of cognitive impairment and blood flow restoration scheme This image was created by the author team using Biorender.com.

The relationship between cerebral perfusion and cognitive function in CS is grounded in the hemodynamic consequences of the condition. Hypoperfusion in critical brain regions, particularly those near the middle cerebral artery (MCA), is a hallmark feature. Prior to revascularization, metrics such as mean transit time (MTT) and bolus arrival time (BAT) are prolonged, reflecting delayed blood flow to areas vulnerable to ischemia (Figure 2). These alterations are associated with cognitive deficits due to decreased oxygen and metabolic substrate delivery, negatively impacting synaptic plasticity and neural networks involved in memory, attention, and processing speed. Prolonged MTT and BAT have been correlated with poorer cognitive performance, suggesting that both the reduction in cerebral blood flow and the inefficiency of hemodynamic transport are relevant contributing factors [8].

Following revascularization, the restoration of cerebral perfusion depends on vascular autoregulatory capacity. In individuals with preserved autoregulation, normalization of blood flow occurs efficiently, facilitating short-term functional and cognitive improvement, observed up to three months post-procedure. In contrast, patients with impaired autoregulation exhibit less effective reperfusion, limiting the neurocognitive benefits of the intervention. Additionally, improvement appears to be greater in patients with pre-existing cognitive dysfunction, suggesting that the brain is more responsive to hemodynamic changes when baseline cognitive deficits are present [9].

*Carotid Endarterectomy* 

Carotid endarterectomy (CEA) confers neurocognitive benefits by enhancing cerebral perfusion and reducing microembolization. Patients with significant carotid stenosis (≥70%) show substantial improvements in memory, attention, and language within one month of the procedure [10]. Bernstein et al. reported that CEA preserves the hippocampal white matter structure and improves cognitive assessments, such as the Montreal Cognitive Assessment (MoCA), compared to patients with asymptomatic carotid stenosis. These cognitive improvements are not solely treatment-related but also reflect the reversal of chronic hypoperfusion effects [11]. Studies suggest that the increase in cerebral blood flow (CBF) following CEA is associated with cognitive gains, particularly in patients with mild cognitive impairment, especially in domains such as attention and working memory [12,13]. Furthermore, patients with impaired cerebral autoregulation or preoperative hypoperfusion tend to experience more pronounced benefits [14].

Complications and Risk Factors

Despite the benefits of CEA, there are risks of neurocognitive complications, such as postoperative cognitive dysfunction (POCD), which affects approximately 19% of patients [15,16]. Cerebral hyperperfusion syndrome, observed in some patients following revascularization, is a critical risk factor for POCD, as it can lead to cerebral edema or cortical neuronal damage. In 1 study, 16% of patients exhibited postoperative cerebral hyperperfusion; those with both hyperperfusion and cerebral microbleeds had a 100% rate of cognitive decline, compared to 20% in patients without hyperperfusion [15]. Intraoperative microemboli may also contribute to early cognitive deterioration, although findings are mixed due to inconsistencies in neuropsychological testing methodologies [13]. Preoperative statin use has been associated with a reduced incidence of POCD, likely due to its neuroprotective effects [16-18]. Variability in cognitive outcomes highlights the need for standardized testing and long-term follow-up to assess the cognitive impact of CEA accurately [13,19].

Age-Dependent Outcomes

CEA demonstrates sustained improvements in executive function, memory, and language up to 12 months post-procedure in patients under the age of 80, indicating a strong neuroprotective potential in this demographic [20]. However, cognitive benefits are limited in patients over 80 years of age, particularly in verbal memory, with no significant improvements observed across evaluated cognitive domains. This suggests that aging may disproportionately affect certain cognitive capacities, such as processing speed and reasoning, while sparing others. Reduced neuronal plasticity and increased vulnerability to surgical stress in older adults may be contributing factors [18,21].

CEA in Polyvascular Disease and Comorbid Conditions

In patients with polyvascular disease undergoing simultaneous CEA and coronary revascularization, the incidence of POCD is high, affecting 56% of those without preoperative cognitive impairment and 71% of those with pre-existing deficits [22]. This elevated risk is attributed to factors such as prolonged anesthesia, systemic inflammation, and cerebrovascular stress. Additionally, diabetic patients are particularly vulnerable to POCD due to microvascular disease and impaired cerebrovascular reactivity [19,23].

Patient Follow-Up

In the medium term, CEA significantly improves cognitive functions, such as memory and executive function, particularly in patients with severe stenosis, with these benefits maintained at six months. These improvements correlate with the restoration of cerebrovascular reactivity, emphasizing the role of hemodynamic restoration in cognitive recovery [24]. Neurophysiological markers, such as EEG characteristics and cognitive event-related potentials (P300), may predict cognitive improvement, assisting in patient selection and identifying pre-existing cortical dysfunction. For instance, increased beta oscillation power in frontal leads and greater P3 amplitude in the P300 component have been associated with post-CEA cognitive improvement. Additionally, heart rate variability and spectral EEG analysis may help predict cognitive trajectory in patients undergoing CEA [24,25]. Nevertheless, prolonged cerebral hyperperfusion remains a risk factor for long-term cognitive decline in some patients, although its effects often diminish over time [20,26].

Carotid Angioplasty With Stent

CAS has demonstrated positive effects on cognitive function in patients with carotid stenosis. Among individuals with asymptomatic stenosis ≥70%, significant improvements were observed in memory, attention, and executive function at 12 months post-procedure, as measured by the MoCA (p < 0.05) [25]. In patients with internal carotid artery occlusion, CAS alleviated cerebral ischemia, resulting in improved scores on both the Mini-Mental State Examination (MMSE) and MoCA during a one-year follow-up period (p = 0.034) [26]. Lin et al. reported a correlation between enhancements in verbal memory and increased cerebral perfusion [27]. However, factors such as advanced age and symptomatic carotid stenosis were associated with subsequent cognitive decline.

MoCA scores improved significantly following CAS, with benefits in memory, attention, and executive function sustained at 12 months. Lin et al. found that improvements in verbal memory were correlated with increased cerebral perfusion, particularly in the middle cerebral artery (MCA) territory, in patients with asymptomatic stenosis ≥70%. After an average of 2.3 years post-treatment, significant reperfusion was observed in this region, evidenced by reductions in relative mean transit time and time to peak (p = 0.03, 0.03, respectively) in the MCA territory, indicating region-specific perfusion changes. Furthermore, a significant increase in fractional anisotropy (FA) - a measure of structural connectivity - was observed in the deep white matter and posterior corpus callosum ipsilateral to the stented hemisphere in the CAS group, reinforcing the importance of cerebral perfusion in cognitive improvement [27].

Both CAS and CEA provide comparable benefits in preventing cerebrovascular events, with no significant differences in early outcomes. At 12 months, both techniques yield improvements in executive function [25], and in the long term, cognitive enhancements are attributed to advances in technique. Nonetheless, short-term outcomes do not reveal statistically significant differences between CAS and CEA [28]. Moreover, CAS combined with intensive medical therapy has demonstrated more pronounced improvements in memory compared to medical therapy alone [27].

Although CAS generally improves cognitive function, certain perioperative risks, such as episodes of hemodynamic depression and intraoperative embolic lesions, may influence outcomes. However, a 24-month follow-up study reported no cases of POCD associated with these events [26,29]. Associations have been found between early postoperative memory and attention deficits and neurochemical markers such as neuron-specific enolase (NSE) and S100β protein (9.0%, p = 0.001). Elevated S100β levels at 24 hours post-surgery were associated with poorer performance in attention and reaction tasks (r = 0.476; p = 0.041) and a higher number of errors in the voluntary attention test (r = 0.449; p = 0.032). Additionally, a significant inverse relationship was observed between NSE levels and performance on the Bourdon test, with a higher number of letters processed correlating with lower NSE levels (r = -0.642; p = 0.014) [30].

CAS is associated with greater cognitive benefits than medical therapy alone, particularly in patients with severe symptomatic carotid stenosis, where it significantly outperforms medication in enhancing memory, attention, and language at six months [31]. In a study conducted in an Indian population, significant improvements in memory and visuospatial functions were observed following CAS, especially among patients with left-sided carotid stenosis [32]. Nevertheless, randomized controlled trials are necessary to draw definitive conclusions regarding the comparative impact of revascularization techniques versus optimal medical management [33]. Table 1 presents a comparative summary of the advantages and disadvantages of both techniques.

**Table 1:** Comparison between carotid endarterectomy and carotid angioplasty with stenting in the treatment of cognitive decline Authored by the investigators, this study presents the main findings reported in the literature for each surgical revascularization technique.

	Carotid Endarterectomy	Carotid Angioplasty With Stenting
Benefits	Significant cognitive improvement in the medium and long term.	Sustained improvement in cognitive function over the long term.
	Particularly effective in patients with pre-existing cerebral hypoperfusion disorders.	More effective when combined with medical therapy.
	Enhances working memory.	Improves visuospatial, executive, and motor domains.
Drawbacks	High risk of developing postoperative cognitive decline.	High risk of intraoperative microembolic complications.
	Greater risk of intraoperative microembolic events.	
	Not beneficial for octogenarian patients.	
	Higher complications due to intraoperative arterial clamping.	

Combined Therapies and Medications

The intraoperative administration of nimodipine in elderly patients undergoing CEA has shown positive effects on cognition. In a study involving 82 patients, those who received nimodipine achieved significantly higher MoCA scores and exhibited a lower incidence of POCD (17% vs. 27%) [34]. These statistically significant improvements (p < 0.05) suggest a positive impact on daily cognitive function, potentially related to enhanced cerebral oxygen metabolism and reduced levels of S100β protein, a biomarker of brain injury. However, further research is needed to determine whether the reduction in S100β levels is associated with long-term cognitive benefits or merely reflects an acute postoperative response.

Several studies have assessed the neuroprotective effects of dexmedetomidine during carotid procedures. In patients undergoing CEA, dexmedetomidine significantly improved MMSE and MoCA scores at 48 and 72 hours postoperatively compared to control groups. While these improvements were statistically significant, additional evidence is required to establish their clinical relevance. The neuroprotective effect has been attributed to the reduction of inflammatory markers (TNF-α and IL-6) and increased levels of brain-derived neurotrophic factor, which is crucial for neuronal plasticity [35]. Moreover, dexmedetomidine reduced preoperative anxiety and provided adequate sedation without adversely affecting postoperative cognitive performance [36]. Nevertheless, the neuroprotective benefits may stem from both anti-inflammatory effects and sedation, suggesting that other sedatives may not yield similar outcomes [37].

Further studies have explored the effects of dexmedetomidine in older patients undergoing minimally invasive coronary revascularization surgery. Results indicated a lower incidence of POCD at seven days post-surgery and higher intraoperative cerebral oxygen saturation [38], highlighting the potential of dexmedetomidine to mitigate postoperative cognitive deficits in vulnerable populations, such as the elderly.

Overall, the evidence supports the use of nimodipine and dexmedetomidine as promising pharmacological interventions to improve cognitive outcomes and provide neuroprotection in elderly patients undergoing high-risk surgical procedures. However, potential side effects, such as hypotension and bradycardia, must be considered, and future studies should explore the long-term effects and underlying mechanisms responsible for these cognitive benefits.

Neurocognitive Changes

Long-term cognitive outcomes following carotid revascularization procedures, such as CEA and CAS, show cognitive improvements in patients with severe carotid stenosis, particularly in attention, verbal fluency, and memory within one year post-procedure, alongside potential risks of cognitive decline. However, advanced age and symptomatic stenosis increase the risk of cognitive deterioration, indicating that outcomes vary according to individual and procedural characteristics [39,40].

Kohta et al. reported that carotid revascularization enhances functional connectivity in key regions such as the medial prefrontal cortex and the left lateral parietal cortex, which was associated with significant improvements in cognitive scores (MoCA: 25.3 vs. 26.8, p = 0.02; frontal assessment battery: 14.4 vs. 15.6, p = 0.01) [41]. Additionally, patients with a lower burden of white matter hyperintensities demonstrated increased connectivity between the left lateral parietal cortex and the precuneus, right lingual gyrus, and right intracalcarine cortex. These changes positively correlated with improvements in MoCA scores (20.1 vs. 23.9, p = 0.0001) [41].

Cognitive improvements following carotid revascularization were more pronounced in patients with pre-existing cognitive impairment. Relander et al. found that CEA improved working memory, especially in patients with neurovascular compromise such as reduced cerebrovascular reactivity or diminished cerebral blood flow. In this subgroup, a significant increase in functional connectivity was observed between the left lateral parietal cortex and the right intracalcarine cortex, right lingual gyrus, and precuneus, correlating with improved MoCA scores [42,43].

CAS has demonstrated greater benefits in visuospatial, executive, and motor functions, whereas CEA has been more effective in improving working memory. This may be due to differences in cerebral blood flow restoration, with CAS potentially providing better reperfusion in areas critical for visuospatial and executive functioning, while CEA may be more closely linked to improvements in working memory through targeted restoration in specific brain regions [40]. Nonetheless, most studies report no significant long-term differences between the two techniques. Furthermore, Yoshida et al. noted that patients with a lower preoperative white matter hyperintensity burden experienced more consistent cognitive improvements following CEA [44].

Age and pre-existing conditions significantly influence the outcomes of carotid revascularization. In patients over 80 years of age, both CEA and CAS are safe for preventing ipsilateral strokes; however, they may be accompanied by reductions in functional capacity and independence, possibly related to frailty and comorbidities. A study involving patients aged 80 and above found that although mortality rates were higher in this group, the incidence of vascular events was comparable to that in younger patients. This suggests that CEA and CAS are both safe and effective treatments for carotid stenosis in elderly individuals, particularly for preventing ipsilateral ischemic stroke [45]. Conversely, younger patients and those with lower baseline MoCA scores derived greater cognitive benefits from revascularization [46].

Neurocognitive changes following carotid procedures are multifactorial and influenced by vascular biomarkers. Parameters such as the ankle-brachial index (ABI) and the cardio-ankle vascular index have emerged as predictors of cognitive decline, even years after surgery. A low ABI has been associated with poorer cerebral perfusion and a higher embolic burden, suggesting that these biomarkers may serve as useful tools for predicting cognitive function and its potential decline in patients undergoing carotid revascularization [47].

Advances in the Management of Carotid Stenosis

The management of carotid stenosis remains controversial and is highly dependent on individual patient characteristics, ranging from optimal candidate selection to the potential utility of inflammatory biomarkers for risk prediction, as well as the chosen treatment modality. Although carotid revascularization through CEA or CAS may benefit specific high-risk subgroups, such as patients with microembolisms, silent infarcts, plaque ulceration, or intraplaque hemorrhage, there is insufficient consensus to support its widespread use in asymptomatic patients. Indeed, revascularization carries inherent risks, including postoperative complications and the potential for restenosis, which may offset the observed benefits. In asymptomatic patients, optimized medical therapy may be sufficient, particularly in those with mild to moderate stenosis. Therapeutic decisions should consider not only clinical and imaging findings but also patient preferences, cultural background, and social context, all of which introduce substantial variability in observed outcomes [48].

Inflammatory biomarkers, such as the neutrophil-to-lymphocyte ratio (NLR) and the platelet-to-lymphocyte ratio (PLR), have shown prognostic potential in carotid artery disease. An elevated NLR is associated with markers of atherosclerosis (e.g., intima-media thickness, plaque presence) and with POCD following CEA. The PLR has been linked to complications such as stroke, coronary syndromes, and mortality. However, their clinical applicability remains limited due to a lack of validation in large-scale studies and the absence of standardized thresholds, necessitating further research to evaluate their role in risk stratification and personalized treatment [49].

Resting-state functional magnetic resonance imaging (fMRI) has proven useful in assessing brain reorganization following CEA. One study reported significant cognitive improvements (MMSE scores) along with enhanced functional connectivity within the default mode network, particularly in the medial prefrontal cortex and other key brain regions. These changes may be associated with improvements in memory and attention, though further studies are needed to determine whether such effects are sustained long-term or are only transient [50].

The monitoring of microembolic signals (MES) via transcranial Doppler has emerged as a relevant risk marker for cognitive impairment. The presence of MES is associated with lower MoCA scores and an increased risk of cognitive decline. A retrospective study found positive MES in 47% of patients with cognitive impairment, compared to only 18.4% in those with normal cognitive function. Multivariate analysis revealed that MES independently increased the risk of cognitive impairment (OR 7.36; p < 0.0001), supporting their utility in guiding interventions and follow-up in high-risk patients [51].

Although MES, inflammatory biomarkers, and fMRI are typically analyzed independently, their integration may enhance risk stratification and patient selection for revascularization. Combining MES monitoring with functional MRI and biomarker analysis could provide a more comprehensive assessment of risks and benefits in selected patients.

Personalized Considerations in Carotid Revascularization for Cognitive Impairment

Advanced age: In patients over 80 years of age, the severity of atherosclerotic plaque is associated with increased intraoperative mortality risk, necessitating a thorough evaluation that includes plaque burden, frailty, and preoperative cognitive status. Although cognitive benefits may be limited in this population, especially among those with significant comorbidities, CEA and CAS remain safe and effective options for preventing ipsilateral cerebrovascular events. Recent studies suggest that while octogenarians may not consistently exhibit cognitive improvements, vascular outcomes are comparable to those in younger patients, supporting the utility of revascularization in select elderly individuals with severe carotid stenosis [18,25,45].

Laterality and cognitive outcomes: The laterality of carotid artery disease significantly influences cognitive outcomes. Left-sided carotid occlusion is associated with a higher risk of cognitive decline, particularly in language function, at 12 months postoperatively. This impairment is proportional to the degree of occlusion and correlates with poorer cognitive recovery [23,25]. In contrast, revascularization may enhance cognitive function in the contralateral hemisphere when perfusion is preserved, potentially due to improved collateral circulation [29]. However, no significant cognitive improvements have been observed in the ipsilateral hemisphere following revascularization, suggesting that factors such as cerebral hemodynamics and plaque burden also modulate postoperative outcomes.

Procedural complications: Post-revascularization hyperperfusion, which may lead to intracerebral hemorrhage or edema, is a major contributor to POCD, with incidence ranging from 6% to 30% following CEA [16,23]. Patients with intraoperative hyperperfusion exhibit a markedly increased risk of POCD (OR: 35.68; 95% CI: 16.64-76.51; p < 0.00001) [16]. Identified risk factors include intraoperative hyperperfusion, prolonged carotid clamping duration (mean difference: 5.25 min; 95% CI: 0.87-9.63; p = 0.02), diabetes mellitus (OR: 1.70; 95% CI: 1.07-2.71), and degree of carotid stenosis (OR: 5.06; 95% CI: 0.86-9.27) [16,23]. In contrast, preoperative statin use has been associated with a reduced risk of cognitive impairment (OR: 0.56; 95% CI: 0.41-0.77; p = 0.0004) [16,23]. Other potential complications include myocardial infarction, cranial nerve injury, cervical hematoma, postoperative arrhythmias, and blood pressure instability [52].

Comorbidities: Comorbidities complicate decision-making regarding carotid revascularization. Patients with polyvascular obstructive disease have a higher risk of adverse outcomes [19]. While essential hypertension has not been clearly linked to postoperative cognitive decline, diabetes mellitus represents a significant risk factor due to its effects on microvascular disease, which compromise cerebrovascular autoregulation and oxygenation [14]. Furthermore, carotid revascularization has not demonstrated a cognitive benefit in patients with severe cognitive impairment related to cerebrovascular events or preexisting deficits [29,36]. The procedure may have limited utility in individuals with significant psychiatric disorders, such as generalized anxiety disorder or major depressive disorder, as these conditions may worsen postoperatively, affecting rehabilitation adherence and increasing the risk of cognitive dysfunction. This observation aligns with findings that postoperative anxiety and depression significantly increase the risk of POCD (p < 0.01) [53].

Weighing risks and benefits

The decision to proceed with carotid revascularization must be based on a careful evaluation of its risks and benefits. While many studies report short-term cognitive improvements, the durability of these effects remains uncertain. Heterogeneity in outcomes may be explained by preoperative factors, such as localized or global cerebral dysfunction, which influence cognitive trajectories after surgery. For example, patients undergoing CEA have been observed to experience more marked improvements in executive function compared to those treated with CAS, although these differences tend to diminish over time. Despite CEA’s efficacy in stroke prevention, its long-term impact on dementia risk appears to be neutral. These findings highlight the importance of identifying predictive factors, such as preexisting structural brain abnormalities or systemic inflammatory markers, which may help determine which patients are most likely to benefit cognitively from surgery [43,54].

Intraoperative mortality and the risk of ischemic events or microembolization may contribute to long-term progressive cognitive decline. Perioperative micro embolisms can cause white matter hyperintensities, which are associated with an increased risk of vascular dementia and long-term microangiopathy. These potential adverse effects underscore the need for careful patient selection and an individualized analysis of risks and benefits when considering carotid revascularization as a therapeutic strategy [10,26,28,35]. Microembolization may result in chronic cerebral hypoperfusion, contributing to cognitive decline and progression of POCD in some patients, while others may experience cognitive improvements following surgery.

Future guidelines

The use of carotid revascularization to improve cognitive function remains a subject of debate due to the lack of standardization in research protocols and the diversity of cognitive assessment tools employed [9,33]. Many commonly used scales, such as the MoCA, lack the specificity needed to detect cognitive deficits related to vascular dysfunction, particularly in older adults [12]. This limitation hampers the accurate measurement of cognitive benefits following carotid revascularization. While some studies employing more refined methodologies report significant improvements, most research relies on global assessment tools that fail to capture domain-specific cognitive impairments, making it difficult to compare findings across studies [30,36,55]. Research focused on specific cognitive domains remains limited.

Imaging findings related to carotid revascularization are inconclusive. Although improved perfusion in previously hypoperfused brain areas has been documented postoperatively, definitive clinical benefits have not been established, largely due to the potential for complications. Pre-existing lesions, such as white matter hyperintensities, cortical atrophy, and lacunar infarcts, are often negative predictors of postoperative clinical improvement [44]. Further investigation is necessary to determine whether particular lesion types may predict more favorable or adverse outcomes, as underlying brain pathology may influence treatment responsiveness.

Although medications such as statins, nimodipine, and dexmedetomidine have demonstrated neuroprotective effects against postoperative cognitive decline, no standardized therapeutic regimen currently exists. Findings are inconsistent, with variations in dosing and study populations complicating the development of uniform protocols. A gender-based approach is also essential, as evidence suggests that men may be less susceptible to cognitive decline, likely due to hormonal, vascular, and neuroprotective differences [12,16,17,29]. Some studies indicate that combining statin therapy with surgery may enhance cognitive outcomes, while others report no significant effects. Furthermore, the cognitive impact of Asperger’s syndrome following revascularization remains unclear and warrants additional research, suggesting that broader consideration of atypical neurodevelopmental profiles may be more appropriate [48].

Ongoing research into the pathophysiology of intraoperative adverse events related to cerebral hyperperfusion in carotid revascularization, particularly using murine models, has begun to elucidate the roles of inflammatory responses, microvascular changes, and oxidative stress. These studies are crucial for developing strategies to mitigate complications and improve procedural outcomes. In vulnerable populations, such as patients with dementia, carotid revascularization techniques have shown limited benefit, highlighting the need for therapeutic approaches that enhance cerebral perfusion while addressing underlying neurodegenerative processes with a vascular component - a potential benefit that has already been partially validated [54,56].

There is substantial methodological heterogeneity in studies evaluating cognitive benefits after carotid revascularization. Establishing a clear definition of cognitive improvement is essential, using specific parameters such as a minimum MoCA score increase or improvements in defined cognitive domains. The duration of cognitive improvements should also be considered when formulating procedural recommendations [11,28,54,56].

## Conclusions

Carotid stenosis impairs cognitive function through chronic cerebral hypoperfusion and silent microembolisms, and it should be considered a key component of the clinical presentation. This underscores the importance of systematically including cognitive assessments in the preoperative evaluation, using tools such as the MoCA and structural neuroimaging biomarkers to stratify risk and guide personalized therapeutic decisions. Although both CEA and CAS have demonstrated cognitive benefits in specific domains, the absence of clear superiority - owing to study heterogeneity and patient variability -necessitates an individualized approach. The choice of procedure should consider surgical risk, vascular anatomy, and the patient’s cognitive reserve.

To optimize cognitive outcomes, a comprehensive strategy is required that includes not only safe revascularization but also rigorous perioperative management, encompassing pharmacological neuroprotection (e.g., nimodipine, dexmedetomidine), multimodal rehabilitation, and control of vascular risk factors. Future clinical guidelines should standardize cognitive assessment protocols, establish robust risk stratification models, and develop long-term follow-up schemes that integrate stroke prevention with both functional and neurocognitive recovery.
